# Authors’ response to: How to manage ventricular arrhythmia in patients with viral myocarditis

**DOI:** 10.1093/ehjopen/oeae006

**Published:** 2024-02-23

**Authors:** Andrea Villatore, Giovanni Peretto

**Affiliations:** Disease Unit for Myocarditis and Arrhythmogenic Cardiomyopathies, IRCCS San Raffaele Scientific Institute, Via Olgettina 60, 20132 Milan, Italy; School of Medicine, Vita-Salute San Raffaele University, Via Olgettina 60, Milan 20132, Italy; Disease Unit for Myocarditis and Arrhythmogenic Cardiomyopathies, IRCCS San Raffaele Scientific Institute, Via Olgettina 60, 20132 Milan, Italy; School of Medicine, Vita-Salute San Raffaele University, Via Olgettina 60, Milan 20132, Italy; Department of Cardiac Electrophysiology and Arrhythmology, IRCCS San Raffaele Scientific Institute, Via Olgettina 60, 20132 Milan, Italy

**Authors' response to the commentary of Kataoka et al., https://doi.org/10.1093/ehjopen/oeae005.**

## To the editor

We thank Drs Kataoka and Imamura for their commentary on our recent original article about arrhythmic viral myocarditis.^1^

The first point raised discusses the biological relevance of ventricular arrhythmias (VA) in patients with viral myocarditis. To deeply characterize the arrhythmic profile of this group, we considered all grades of VA, including the minor ones (non-sustained VA and/or ectopies). However, our multivariable analysis demonstrated that only the major ones (sustained VA and/or ventricular fibrillation) were predictive for adverse outcomes.

Second, an important clarification should be made about early VA, which were defined merely on a temporal criterion (i.e. <24 h from hospitalization), without any reference to the true onset of inflammatory heart disease. As previously demonstrated by our group,^2^ polymorphic VA are an expression of a diffuse inflammatory substrate and, specifically in viral aetiology, virus-mediated pro-arrhythmic mechanisms (e.g. direct cytopathic effect and gap junction disruption). On the contrary, monomorphic VA are related to dense scar, as a consequence of replacement fibrosis. Therefore, while polymorphic VA suggest an acute or early-stage process, monomorphic VA should point to a longer-standing cardiomyopathic damage with a possible delay until medical attention.

As regards to heart failure trajectories, in our study, left ventricular ejection fraction (LVEF) at presentation failed to predict late adverse outcomes. Not surprisingly, many patients with polymorphic VA had fulminant myocarditis showing prompt recovery of systolic function before discharge. On the other hand, in the chronic stage of myocarditis, a reduction in LVEF may be expected at a longer timepoint than the median follow-up in this study (24 months).

One last relevant point regards the prediction of sudden cardiac death in myocarditis. While implantable cardiac defibrillator (ICD) implantation was traditionally deferred until resolution of the acute episode following presentation with major VA, an earlier timing is now recommended by the European Society of Cardiology guidelines.^3^ As supported by our findings in viral myocarditis, presentation with monomorphic VA, biopsy-proven fibrosis, and low-load parvovirus B19 genome may further strengthen the recommendation to an early ICD implant. Conversely, a watchful waiting strategy is more appropriate for patients presenting with polymorphic VA and no associated high-risk features.

## Data Availability

No new data were generated or analysed in support of this research.
